# To Feed or Not to Feed: A Critical Overview of Enteral Feeding Management and Gastrointestinal Complications in Preterm Neonates with a Patent Ductus Arteriosus

**DOI:** 10.3390/nu12010083

**Published:** 2019-12-27

**Authors:** Silvia Martini, Arianna Aceti, Silvia Galletti, Isadora Beghetti, Giacomo Faldella, Luigi Corvaglia

**Affiliations:** Neonatal Intensive Care Unit, S. Orsola-Malpighi University Hospital, Department of Medical and Surgical Sciences, University of Bologna, 40138 Bologna, Italysilvia.galletti4@unibo.it (S.G.); i.beghetti@gmail.com (I.B.); giacomo.faldella@unibo.it (G.F.); luigi.corvaglia@unibo.it (L.C.)

**Keywords:** patent ductus arteriosus, preterm infants, enteral feeding, enteral nutrition, gastrointestinal complications, necrotizing enterocolitis, feeding intolerance, indomethacin, ibuprofen, paracetamol, human milk

## Abstract

The management of enteral feeds in preterm infants with a hemodynamically significant patent ductus arteriosus (hs-PDA) is a major challenge for neonatologists due to the fear of gastrointestinal (GI) complications. This review aims to analyze the available evidence on the complex relation between the presence and management of PDA, enteral feeding practices, and GI outcomes in the preterm population. There is limited evidence, based on small and heterogeneous trials, that hs-PDA may affect the splanchnic hemodynamic response to enteral feeds. While the presence of PDA seems a risk factor for adverse GI outcomes, the benefits of feeding withholding during pharmacological PDA treatment are controversial. The lack of robust evidence in support of or against a timely feeding introduction or feeding withholding during pharmacological PDA closure in preterm neonates does not allow to draw any related recommendation. While waiting for further data, the feeding management of this population should be carefully evaluated and possibly individualized on the basis of the infants’ hemodynamic and clinical characteristics. Large, multicentric trials would help to better clarify the physiological mechanisms underlying the development of gut hypoperfusion, and to evaluate the impact of enteral feeds on splanchnic hemodynamics in relation to PDA features and treatment.

## 1. Introduction

A persistent patent ductus arteriosus (PDA) is a common condition among preterm neonates. A complex interplay of factors, such as increased sensitivity to the vasodilating effects of prostaglandin E2 (PGE2) and nitric oxide [1], a less muscular and thin-walled ductal structure, early adrenal insufficiency, and impaired platelet function, contribute significantly to prolonged ductal patency in the preterm population [2]. The rate of spontaneous ductal closure in very low birth weight infants is inversely proportional to gestational age at birth, with a median time to closure ranging between 6 days in infants ≥ 30 weeks and 71 days < 26 weeks’ gestation [3].

As pulmonary vascular resistances decline over the first days after birth, the blood flow proportion that is diverted by the PDA from systemic to pulmonary circulation progressively increases. While the ensuing pulmonary overflow predisposes to the development of pulmonary congestion and increases the risk of pulmonary hemorrhage and bronchopulmonary dysplasia, the blood flow “steal” from the descending aorta to pulmonary arteries may exceed the physiological compensatory mechanisms aimed at increasing left cardiac output, with subsequent reduction of end-organ perfusion. As such, prolonged ductal patency is associated with higher mortality rates [4] and several adverse outcomes, including intraventricular hemorrhage, periventricular leukomalacia, impaired renal function, and necrotizing enterocolitis (NEC) [5]. To which extent these conditions are attributable to the hemodynamic consequences of PDA has not been fully elucidated; however, the development of gastrointestinal (GI) complications in preterm infants with a hemodynamically significant (hs-) PDA is a common fear among neonatologists, and represents a major challenge for the management of enteral feeds in this delicate population.

Hence, this narrative review is aimed to analyze and discuss the available literature investigating the complex relation between the presence and treatment of PDA, enteral feeding practices, and GI outcomes in very preterm infants.

## 2. Literature Review

A literature search was conducted for studies published before 15 November 2019 in PubMed (http://www.ncbi.nlm.nih.gov/pubmed/), the Cochrane Library (http://www.cochranelibrary.com/), and Embase (http://store.elsevier.com/en_US/info/30800006). Specific strings were built up by combining all of the terms related to PDA and enteral feeding; PubMed MeSH terms, free-text words, and their combinations obtained through the most proper Boolean operators were used. The same criteria were used for searching the Cochrane Library and Embase. Reference lists of the resulting papers were also checked for further literature implementation.

## 3. Discussion

### 3.1. Hemodynamic and GI Effects of Pharmacological Treatments for PDA Closure

In order to avoid a prolonged exposure to a trans-ductal systemic-to-pulmonary blood shunting, a targeted pharmacological PDA treatment, based on the infants’ clinical and echocardiographic characteristics, is widely adopted as the first-line approach for PDA closure in very and extremely preterm neonates. To date, indomethacin, ibuprofen, and, more recently, paracetamol are the main pharmacological agents used for PDA closure. A number of possible adverse GI effects have been reported in association with these drugs, and therefore should be taken into account in the choice of PDA pharmacological treatment.

Indomethacin is a non-steroidal anti-inflammatory drug (NSAID) that, via non-selective cyclooxygenase (COX) 1 and COX-2 inhibition, hinders the synthesis of PGE. Indomethacin was introduced for pharmacological PDA treatment in the mid-1970s; since then, this drug has long been the treatment of choice, despite its association with renal and GI complications, secondary to the potent vasoconstrictor effects of indomethacin on splanchnic circulation [6]. A significant decrease of blood flow velocity (BFV) in the superior mesenteric artery (SMA) has been reported following the administration of indomethacin boluses [7,8]. This, along with the impairment of mesenteric hemodynamics ensuing from the systemic ductal steal, and the noxious effects of PGE synthesis inhibition on gut mucosa [9], can add to the risk of developing such ischemic-based complications as NEC or spontaneous intestinal perforation (SIP). Increased SIP rates have been reported following indomethacin treatment for PDA closure [10], and in case of concurrent indomethacin and corticosteroid treatment [11], which, therefore, should be avoided. The hemodynamic perturbances related to indomethacin, however, can be effectively attenuated by its continuous infusion over 36 h, which, conversely to bolus injections, does not significantly decrease renal and mesenteric perfusion [8].

Ibuprofen, which also belongs to the NSAID group, causes a rapid and reversible non-selective competitive inhibition of both COX-1 and COX-2 iso-enzymes. This drug emerged as a valid alternative to indomethacin from the mid-90s, demonstrating equal efficacy on PDA closure but lower risks of NEC and transient renal insufficiency [12]. Unlike indomethacin, ibuprofen, either oral or intravenous, has not been shown to affect mesenteric blood flow significantly [13,14]. As to the effect on splanchnic tissue oxygenation (SrSO_2_), evaluated by near infrared spectroscopy (NIRS), Bhatt et al. observed a greater, although not significant, reduction of SrSO_2_ in preterm infants with PDA treated with indomethacin compared to ibuprofen. Despite some concerns having been raised on a possible association between oral ibuprofen and the occurrence of GI bleeding, NEC [15], and bowel perforation [16], due to the high osmolality of the oral formulation [17], a recent meta-analysis has provided low-quality evidence of a reduced risk of NEC in association with oral compared to intravenous ibuprofen, whereas no difference in the risk of SIP and GI bleeding was observed between oral and intravenous routes [12].

Paracetamol inhibits the peroxidase site of prostaglandin H2 synthase and has also been associated with selective COX-2 inhibition [18]. In 2011, an incidental observation of PDA closure after the use of paracetamol in a few infants who had previously failed ibuprofen treatment led Hammerman et al. to consider off-label use of this drug for PDA [19]. Since then, further observational studies have supported this preliminary evidence, following which the use of paracetamol for ductal closure in preterm infants has progressively increased. According to recent meta-analyses [20,21], the efficacy of paracetamol in achieving ductal closure is similar to that of ibuprofen, especially if used in infants ≥ 28 weeks’ gestation, with a postnatal age < 7 days, and if used as first-line therapy rather than after NSAID failure [21]. Moreover, due to its different mechanism of action, paracetamol is associated with a poorer anti-platelet activity compared to non-selective NSAIDs, which also inhibit COX-1-mediated platelet aggregation [18]. Due to the immature metabolism of preterm infants and of the used dosage (15 mg/kg every 6 h), which is higher than that adopted in term neonates for pain and fever control, the risk of hepatotoxicity related this pharmacological approach is theoretically high [22]. To date, no evidence of hepatotoxicity has been reported in association with paracetamol use for ductal closure; due to the relatively recent introduction of this drug for this therapeutic purpose, however, further data are needed to better define this dosage safety profile, including possible GI effects, in preterm infants with PDA.

A recent meta-analysis has investigated the possible association between the use of indomethacin, ibuprofen, paracetamol, and placebo for ductal closure and the incidence of NEC. Continuous infusion of intravenous ibuprofen was associated with the lowest NEC incidence, whereas standard or high doses of intravenous ibuprofen and standard doses of intravenous indomethacin ranked worse than placebo or no treatment, but these differences failed to reach statistical significance [23].

### 3.2. Hemodynamic and GI Effects of Surgical PDA Ligation

Surgical closure of a hs-PDA is considered a rescue treatment when medical treatment fails or there is a contraindication for cyclooxygenase inhibitor prescription. However, surgical treatment of PDA is related to increased mortality and morbidity; actually, in a large Canadian cohort of preterm infants with GA ≤ 32 weeks, surgery for PDA, compared to conservative and pharmacological management, was associated with a significantly higher risk of a composite adverse outcome, including mortality or any of the following: intraventricular hemorrhage grade 3 or 4, periventricular leukomalacia, severe retinopathy of prematurity, bronchopulmonary dysplasia, or NEC stages 2 or 3 [24]. Similar data were reported in a cohort of extremely low birth weight infants, where infants treated with primary or secondary surgery for PDA were at increased risk of neurodevelopmental impairment and bronchopulmonary dysplasia compared to those receiving indomethacin. In that study, however, no difference in NEC occurrence was documented between surgical and pharmacological treatment of PDA [25].

Previous studies comparing different timings of PDA ligation reported significantly higher rates of NEC [26] and a greater delay in full enteral feeding (FEF) achievement [27] in preterm infants undergoing surgical closure of PDA after the first 2 or 3 weeks of life, respectively, as a possible consequence of the prolonged exposure to the hemodynamic effects of hs-PDA.

One out of 4–5 infants who require PDA surgical ligation, develops a post-ligation cardiac syndrome (PLCS), characterized by hypotension requiring cardiovascular support and ventilation or oxygenation failure [28]. The increase in systemic vascular resistance which follows PDA surgical ligation, together with a decrease in left ventricular preload, are probably involved in the pathophysiology of PLCS.

While a significant association between PLCS and adverse pulmonary and neurodevelopmental outcomes has been reported [28,29], whether the hemodynamic disturbances which characterize PLCS could constitute a risk factor for GI complications has not been clarified yet. Previous data have shown that the need for a surgical treatment for PDA was associated with an almost one-week increase in the time to achieve FEF in a large cohort of very low birth weight infants [30]. Nevertheless, it is unclear whether this delay is related to the characteristics of PDA leading to surgical closure or to the procedure itself.

Recently, PDA transcatheter occlusion has been proposed as an alternative to conventional surgical ligation; this technique appears promising in terms of lower occurrence of PLCS [31]. However, the actual efficacy and safety of catheter occlusion vs. standard surgical ligation still need to be assessed in ad hoc randomized controlled trials [32].

### 3.3. Role of Clinical Variables on Ductal Patency and GI Outcomes

The hemodynamic effects of PDA, together with the perturbances of mesenteric perfusion and the adverse effect profiles of the previously discussed pharmacological strategies for PDA closure, are a major concern for the nutritional management of preterm infants. Due to the fear of GI complications, feeding introduction and the subsequent increase of enteral intakes are often delayed in the presence of a hs-PDA, or during pharmacological PDA closure, as suggested by evidence of an increased time to FEF achievement in the presence of PDA, net of other clinical covariates [30,33]. However, neonatal clinical characteristics other than hs-PDA should also be taken into account for the feeding management of preterm infants with a PDA.

Intrauterine growth restriction (IUGR) of placental origin has been associated with higher rates of PDA-related systemic ductal steal in preterm infants within 48 h after birth [34]. During intrauterine life, however, IUGR fetuses adapt to the chronic hypoxia ensuing from placental insufficiency by undergoing a blood flow redistribution that favors cerebral perfusion at the expense of splanchnic circulation. This places the gut tissue at risk of hypoxic–ischemic injury, and thus represents a possible risk factor for the development of NEC, SIP, and feeding intolerance [35,36], especially in placental-related IUGR infants ≤ 29 weeks’ gestation [37], consistently with the detrimental role of gut immaturity on the development of these complications [38]. A decreased SMA–BFV, both at baseline and after an enteral feed, has been described in neonates with antenatal evidence of absent/reversed end-diastolic umbilical flow (AREDF) [39], which is a proxy of insufficient placental circulation [40]; similarly, preterm infants with AREDF showed significantly lower SrSO_2_ values at enteral feeding introduction compared to those with normal antenatal Doppler [41]. Hence, although targeted clinical evidence is lacking, the combination of the hemodynamic effects of PDA and IUGR of placental origin might theoretically contribute to increase the GI risk in preterm neonates, thus requiring an even more careful nutritional management.

The hemodynamic status of the infant and the concomitant use of vasoactive drugs should be also considered for a tailored management of enteral nutrition in this delicate population. To this regard, hypotension, which is a common manifestation of hs-PDA, may contribute to reduce bowel perfusion, and has been associated with a significantly higher risk of NEC development in a recent systematic review [42]. Dopamine and dobutamine are the most widely used inotropes to support blood pressure and cardiac function in preterm infants, including those with a hs-PDA. Due to their effect on α- and β- adrenoreceptors, the possible impact of these drugs on splanchnic circulation has been previously investigated, leading to controversial results. While in 1998 Seri et al. observed no changes in mesenteric blood flow in hypotensive neonates during dopamine treatment [43], a few years later, the same authors described a significant dopamine-induced increase of SMA–BFV in preterm infants pre-treated with indomethacin [44]. Conversely, Zhang et al. reported a fall in SMA velocity in preterm infants whose cardiac output decreased after dopamine treatment [45]. As to the effect of inotropes on neonatal morbidities, no difference in NEC rates between infants treated with dopamine and dobutamine were reported, while no evidence comparing inotropes to no treatment in preterm infants with low systemic perfusion is actually available [46].

A possible correlation between red blood cell (RBC) transfusion and NEC development has been widely debated over the last decades [47]. In this regard, Sellmer et al. have described a case series of full-fed preterm infants with PDA who developed NEC after receiving a RBC transfusion, thus raising concerns on the simultaneous presence of PDA and RBC administration as a possible risk factor for adverse intestinal outcomes [48].

Eventually, the type of milk feeding should be also taken into account. Human milk, either maternal or donor, plays a key role in promoting gut maturation and, compared to preterm formulas, has been associated with several nutritional and clinical benefits in very preterm infants, including lower risk of GI complications [49], earlier FEF achievement [30], and improved neurodevelopment [50]. Hence, in the presence of a hs-PDA, when human milk is not available, a more careful management of enteral nutrition may be advisable.

### 3.4. Enteral Feeding in Infants with PDA

#### 3.4.1. Impact on Hemodynamics Response to Feeds

Mesenteric hemodynamics before, during, and after feed administration can be assessed through Doppler evaluation of blood flow in the SMA and NIRS, which, by providing a continuous, non-invasive monitoring of SrSO_2_, may add useful information to Doppler data for a better understanding of the impact of PDA-related hemodynamic changes on the balance between gut oxygen delivery and consumption at tissue level [51]. The physiological hemodynamic response to an enteral feeding is a post-prandial increase of SMA–BFV, aimed to meet the metabolic intestinal demands, which can be detected from the first feeding exposure [52,53]. Similarly, a rise of SrSO_2_ has been reported after bolus feeding administration in neonatal NIRS studies [54,55]. Only a few animal and human trials, summarized in Table 1, have investigated whether the hemodynamic perturbances associated with hs-PDA and the pharmacological agents adopted for PDA closure may influence the splanchnic response to enteral feeding administration.

In 2008, McCurnin et al. [56] evaluated SMA–BFV and relative vascular resistance (RVR; mean blood pressure/mean SMA velocity) before and 10 and 30 min after enteral feeding in mechanically ventilated preterm baboons with a moderate PDA shunt, defined by a pulmonary-to-systemic blood flow ratio ≥ 2:1, or with a closed ductus. Despite significantly lower pre-prandial blood pressures and systemic blood flows in baboons with a moderate PDA compared with controls, pre-prandial SMA blood flow velocity did not differ between the two groups. However, while an increase in diastolic and mean SMA–BFV and a decrease in SMA–RVR were observed 10 min after feeding in animals with a closed ductus, with a return to baseline values by 30 min, baboons with a moderate PDA showed no significant changes in SMA velocities or RVR after feeding administration. Consistently, SMA–BFVs 10 min after feed were significantly lower, and RVR significantly higher, in this group compared to controls. As the authors speculated, this impaired response to feeding administration may interfere with the ability of mesenteric perfusion to meet the intestinal metabolic demands related to enteral feeds, thus possibly contributing to the development of feeding difficulties.

A similar trial was performed in extremely low birth weight infants by Havranek et al. in 2015 [57]. Pre- and post-prandial time-averaged mean, peak-systolic, and end-diastolic velocity in SMA were measured in preterm infants aged 5–7 days. According to the PDA features, infants were clustered into small (PDA/left pulmonary artery [LPA] ratio < 0.5, *n* = 16), moderate (PDA/LPA ratio ≥ 0.5 but <1, *n* = 13), and large (PDA/LPA ≥ 1, *n* = 9) PDA groups. Although lower baseline SMA–BFVs were observed in infants with a large PDA, a significant between-group difference was observed only in the end-diastolic phase. Lower SMA–BFVs in the large PDA group were also observed 60 min after the administration of a small test feed; however, the between-group comparison did not reach statistical significance.

We recently investigated the 3-h averaged mean values of SrSO_2_ and splanchnic–cerebral oxygen ratio (SCOR), which has been proposed as a marker of gut hypoxia–ischemia [58], at the time of enteral feeding introduction in very preterm infants with hs-PDA, defined by the evidence of a pulsatile trans-ductal shunt pattern and left atrium to aortic root (LA/Ao) ratio ≥ 1.5 (*n* = 11), restrictive PDA, defined by the evidence of a restrictive trans-ductal shunt pattern and LA/Ao < 1.5 (*n* = 11), and no evidence of PDA (*n* = 28) [59]. Continuous SrSO_2_ and SCOR patterns before and after the administration of the first feed were also compared between the study groups. Mean SrSO_2_ and SCOR values were slightly but not significantly lower in infants with hs-PDA compared to the other groups. Similarly, this group showed reduced SrSO_2_ and SCOR patterns before and after feeding administration, but the between-group comparison did not reach statistical significance at any time point. The small study sample of these trials [57,59], however, may have limited the power of the study analysis and the observed non-significant results.

As to the impact of different feeding managements during PDA treatment with either ibuprofen or indomethacin, Yanowitz et al. investigated mesenteric BFV before, 10 min and 30 min after a test feed of 4 mL/kg in very preterm infants with infants with a hs-PDA requiring pharmacological closure during their second week of life [60]. The enrolled infants were initially randomized to ibuprofen or indomethacin, and then to feeding (15 mL/kg/day bolus feeds) or nihil per os (NPO) during the treatment period. The test feed was administered 18–24 h after the last dose of study drug in the NPO group and 3 h after the last trophic feed in the fed group. No significant differences were observed between infants fed and kept NPO in baseline and post-feed SMA–BFV: Both groups had similar and significant increases in systolic, mean, and diastolic SMA velocities by 30 min after the feed, although this rise in SMA–BFV was already appreciable 10 min after the test feed infants compared with those kept NPO. The limited sample size and the uneven percentage of infants treated with indomethacin compared to ibuprofen (94 vs. 6%), caused by the unavailability of the latter drug for most of the enrollment period, are major limitations of this trial. Given the known impact of indomethacin on mesenteric blood flow, the prevalent use of this drug in the enrolled infants may have played a role in the observed results.

#### 3.4.2. Association between PDA, Feeding Practices, and GI Outcomes

Over the past decades, the possible association between the ductal status and GI outcomes in preterm infants has been variously evaluated; a summary of current literature is available in Table 2.

In 2007, Patole et al. [33] investigated the incidence of NEC and the time needed to reach enteral intakes of 150 mL/kg/day in relation to different clinical characteristics. While no significant association was observed between the development of NEC stage > 2 and PDA, the presence of a hs-PDA, defined by the evidence of a pulsatile trans-ductal shunt pattern with an LA/Ao ratio > 1.4 or a ductal diameter > 1.5 mm, was associated with a significant delay in enteral feeding introduction and with a significantly longer interval between the first feed and FEF achievement (odds ratio (OR) 2.80, 95% confidence interval (CI) 1.35–5.81, *p* = 0.006), which further increased in the case of concomitant PDA and sepsis (OR 5.15, 95% CI 2.13–12.45, *p* = 0.001).

Similar results have been observed in a large registry-based cohort of very low birth weight preterm infants (*n* = 1864) by Corvaglia et al., who evaluated the main predictors for FEF achievement, defined as enteral intakes ≥ 150 mL/kg/day [30]. Among the variables included in the study analysis, the echocardiographic evidence of PDA during the first week of life represented a strong independent predictor for a longer time to FEF (OR 1.276, *p* < 0.001). Nevertheless, these data, derived from the local perinatal network registry, did not take into consideration the PDA characteristics (e.g., size, pattern, and other features suggestive for a hs-PDA).

The number of days required to reach enteral intakes ≥ 150 mL/kg/day in relation to the ductal status was also evaluated as a marker for feeding tolerance by Havranek et al. [57]. Preterm infants in the large PDA group (PDA/LPA ≥ 1) required a significantly longer period to achieve FEF compared to those with moderate (PDA/LPA ratio ≥ 0.5 but <1) and small (PDA/LPA ratio < 0.5) or closed ductus (*p* = 0.02). Although the incidence of NEC (Bell’s stage ≥ 2) [61] did reach statistically significant differences among the study groups, death rate secondary to NEC was significantly higher in the large compared to no/small and moderate PDA groups (*p* = 0.04).

We recently examined the overall incidence of SIP, NEC (Bell’s stage ≥ 2 [61]) and feeding intolerance (withholding of enteral feeds for ≥24 h due to the presence of ≥2 of the following: Absent bowel sounds, abdominal distension, bloody stools, persistent bilious or bloody gastric residuals, residual volume > 2 mL/kg or greater than half the volume of the previous feed) [62] during hospital stay in infants with hs-PDA (evidence of a pulsatile trans-ductal shunt pattern and LA/Ao ratio ≥ 1.5, *n* = 11), restrictive PDA (restrictive trans-ductal shunt pattern and LA/Ao < 1.5), and no evidence of PDA who were commenced on enteral feeds within the first 72 h of life, irrespective of their ductal status [58]. No difference in the rates of the above GI complications was observed between the study groups; the time to FEF achievement, defined as enteral intakes ≥ 150 mL/kg/day, was also evaluated, showing similar between-group results.

A survey conducted among neonatal nurses in the United States (US) and in Canada documented that PDA pharmacological treatment is perceived as one of the leading contraindications to enteral feeding in preterm infants [63]. However, whether withholding feeding during pharmacological PDA closure could help preventing the occurrence of GI complications in infants with a hs-PDA is still a matter of debate among neonatologists. To this regard, data are still very scarce. Clyman et al. [64] randomized 177 preterm infants requiring pharmacological PDA closure to receive trophic feeds (*n* = 81: Indomethacin = 80%, ibuprofen = 20%) or remain NPO (fasting group, *n* = 96: Indomethacin = 75%, ibuprofen = 25%) during the drug administration period, and evaluated the time needed to achieve a daily enteral intake of 120 mL/kg/day, compared to an “ideal number” of days to reach this outcome, based on the assumption that the infant would be fasting during the drug administration period. Daily enteral intakes prior to study entry were similar between the two groups. Infants randomized to the feeding arm required fewer days to reach the feeding volume endpoint, although the age at central venous lines removal did not differ between the two study groups, and there was no between-group difference in the incidence of SIP and NEC (defined as Bell’s stage ≥2 [61]) during the first week of life. The authors justified this delay in achieving the study endpoint with the occurrence of complications other than NEC, SIP, and feeding intolerance, but yet related to ductus patency (e.g., respiratory deterioration, hypotension requiring inotropes, PDA ligation).

More recently, similar findings have been reported by Louis et al. [65], who retrospectively investigated the prevalence of NEC stage ≥ 2a [61], feeding intolerance, defined as at least two consecutive gastric aspirates of >50% of previous feed volume or any aspirate containing bile or blood, time to enteral intakes of 120 and 160 mL/kg/day, and length of parenteral nutrition in relation to enteral feeding intakes at the time of indomethacin therapy in preterm neonates with PDA. Based on their enteral feed volume exposure during treatment, the enrolled infants were stratified into the following groups: NPO (*n* = 229); intake ≤ 60 mL/kg/day (*n* = 142); intake > 60 mL/kg/day (*n* = 44). All groups had similar rates of the evaluated outcomes with the exception of days to reach FEF enteral feeds, which were significantly lower in infants fed ≥ 60 mL/kg/day during indomethacin treatment. Nevertheless, no between-group difference in the duration of parenteral nutrition was observed.

The above findings may underlie the following study bias: the feeding amounts that infants in the feeding group received during PDA accounted for the period of minimal enteral feeding required to advance enteral intakes according to the study protocols, thus allowing an earlier achievement of FEF compared to infants who started feeds later.

## 4. Conclusions

The management of enteral feeding in preterm neonates with evidence of a hs-PDA has long been a matter of debate, with widely different opinions among neonatologists worldwide. More than a decade ago, Jhaveri et al. [66] developed a questionnaire to investigate feeding practices in infants with hs-PDA, defined as a moderate-to-large PDA with holodiastolic retrograde flow in the descending aorta, between US and non-US countries: 70% of US neonatologists believed that enteral feedings need to be stopped in the presence of hs-PDA, due to the fear of possible unfavorable GI outcomes, whereas the same percentage of non-US neonatologists held exactly the opposite opinion. This marked difference in willingness to feed infants with hs-PDA played also a role in the large geographic differences in PDA ligation preferences. In 2009, a survey on feeding practices in the Neonatal Intensive Care Units of North America yielded similar results: most of the respondents withheld feeds in infants with hs-PDA and during low-dose dopamine or indomethacin treatment [67]. These data, however, were collected before the routine introduction of ibuprofen and paracetamol for treating PDA; therefore, the neonatologists’ opinion may have been influenced by the side effect profile of indomethacin, including the previously discussed effects of this drug on splanchnic hemodynamics. Hence, an updated investigation of the main approaches to enteral feeds in relation to PDA across different countries would bring useful information to follow-up the actual practices and highlight possible changes.

Currently available literature aimed at investigating the relation between PDA, feeding practices, and possible GI complications, however, has not brought a substantial contribution to disentangle this knot. Although the hemodynamic mechanisms through which hs-PDA can affect mesenteric perfusion have been theoretically established (e.g., ductal steal), their real impact on the hemodynamic response to enteral feeds deserves to be further investigated, as the few available trials are based on small samples and flawed by a significant methodological heterogeneity. First, the adoption of different definitions for hs-PDA and of variable post-prandial time windows for SMA Doppler evaluation are major limiting factors for the generalizability of the trial results. Moreover, most of the available literature does not provide data on the hemodynamic response to feeds in the presence of a significant diastolic reflow in the SMA, which represents a key indicator of abnormal gut perfusion [68] and may therefore identify infants with hs-PDA at highest risk of GI complications.

As to the assessment of a possible association between GI outcomes and different feeding practices in relation to the presence of a hs-PDA and its treatment, current literature is again markedly heterogeneous. While there is consensus on NEC definition, the criteria adopted to define feeding intolerance range from various signs and symptoms to the number of days needed to achieve variables endpoints of enteral intakes (e.g., from 120 to 160 mL/kg/day). The latter parameter, however, may be influenced by multiple factors, including clinical conditions not strictly related to PDA or to the development of gut complications. Moreover, the previously mentioned variability of hs-PDA definitions further hinders the comparability of the available data on gastro-intestinal outcomes, and no data are specifically available on infants with PDA-related SMA reflow.

Hence, the development of a universal feeding approach in preterm infants with either a clinically or hemodynamically significant PDA still seems a long way off from current perspectives. Large, multicentric trials aimed at analyzing the impact of specific PDA features (e.g., presence/absence of diastolic reflow in SMA) on mesenteric hemodynamics with combined Doppler and NIRS evaluations would help to better clarify the physiological mechanisms underlying the development of gut hypoperfusion and to evaluate the impact of enteral feeds on splanchnic hemodynamics in preterm infants with PDA. Likewise, it is fundamental to standardize the definitions adopted not only for hs-PDA, but also for clinical outcomes such as FEF achievement and feeding intolerance. Eventually, the inclusion of multicentric, international cohorts may be useful to address possible differences either in PDA management or in enteral feeding practices, and to achieve adequately powered samples to evaluate the weight of concomitant risk factors for GI complications (e.g., GA, placental-related IUGR, etc.).

The lack of robust evidence in support or against a timely feeding introduction in preterm infants with hs-PDA or feeding withholding during pharmacological PDA closure should not be interpreted as an endorsement of either one feeding approach or the other; while awaiting for further evidence, the enteral nutrition management of this delicate population should be carefully evaluated and individualized on the basis of the infants’ hemodynamic and clinical characteristics.

## Figures and Tables

**Table 1:** Effects of patent ductus arteriosus (PDA) and of the pharmacological agents adopted for its closure on splanchnic hemodynamic response to enteral feeds.

Authors	Study Population	PDA Definition	Outcome	Results
Mc Curnin et al. 2008 [52]	Premature baboons with moderate (*n* = 11, mean postnatal age 11.6 ± 1.3 days), small PDA shunt (*n* = 13, mean postnatal age 11.6 ± 1.0 days), or closed ductus (*n* = 13, mean postnatal age 11.5 ± 1.1 days).	PDA with moderate shunt: Pulmonary-to-systemic output ratio (Qp/Qs) ≥ 2; closed ductus: Qp/Qs ≤ 1.2.	Comparison of diastolic, mean and peak-systolic SMA–BFV, SMA relative vascular resistance (RVR, mean arterial blood pressure divided by mean SMA–BFV), and pulsatility index (PI, a surrogate measure of SMA impedance) before and 10 and 30 min after feed administration between animals with moderate shunt and no PDA.	Compared to pre-prandial values, animals with a closed ductus showed a significant increase in diastolic and mean SMA–BFV, and a significant RVR decrease 10 min after feeding; by 30 min, these parameters returned towards pre-prandial baselines. No change in SMA–BFVs or in RVR at 10 or 30 min after feeding was observed in the moderate shunt group. Pre- and post-prandial PI was significantly higher in moderate shunt compared to closed ductus group.
Havranek et al. 2015 [53]	Preterm infants with birth weight < 1000 g, aged 5–7 days, with large (*n* = 9, mean GA 25.1 ± 1.3 weeks), moderate (*n* = 13, mean GA 25.8 ± 1.4 weeks), or small/no PDA (*n* = 16, mean GA 26.5 ± 1.4 weeks), not treated in the prior 24 h.	Large PDA: Ductal diameter-to-left pulmonary artery (PDA/LPA) ratio ≥ 1; moderate PDA: PDA/LPA ratio 0.5–0.9; small PDA: PDA/LPA ratio <0.5.	Comparison of time-averaged mean, peak-systolic, and end-diastolic SMA–BFV before and 60 min after a test feed (mean volume: 2.4–3 mL) among the 3 study groups.	Attenuation of postprandial SMA–BFVs at 60 min postprandially in infants with large PDA, although the comparison to other PDA categories did not reach statistical significance (*p* = 0.08). Pre-prandial SMA–BFV tended to be lower in the large PDA group, with significant between-group difference in the end-diastolic phase (*p* = 0.002).
Martini et al. 2019 [55]	Preterm infants < 32 weeks’ gestation with hs-PDA (*n* = 11, median GA 29.4 [IQR 27.4–30.4] weeks), restrictive PDA (*n* = 11, median GA 29.5 [IQR 28.7–30.7] weeks), and with no PDA evidence (*n* = 28, median GA 29.7 [IQR 27.7–31] weeks), receiving enteral feeds within the first 72 h of life, irrespective of PDA status.	Pulsatile/hs-PDA: Pulsatile trans-ductal shunt pattern and left atrium-to-aortic root (LA:Ao) ratio ≥ 1.5; restrictive PDA: Restrictive trans-ductal shunt pattern and LA/Ao ratio < 1.5.	3-h averaged mean values and continuous patterns of splanchnic tissue oxygenation (SrSO_2_) and splanchnic–cerebral oxygenation ratio in response to the first enteral feed administration.	Lower SrSO_2_ and SCOR mean values in the hs-PDA group, although the between-group comparison did not reach statistical significance. No significant difference in SrSO_2_ and SCOR patterns in response to enteral feeding administration.
Yanowitz et al. 2014 [56]	Preterm infants <31 weeks’ gestation undergoing PDA closure with indomethacin or ibuprofen, randomized to receive 15 mL/kg/day bolus feeds (*n* = 16, mean GA 26.3 ± 1.8 weeks) or to be kept NPO (*n* = 18, mean GA 26.4 ± 2.0) during treatment.	Ultrasound evidence of a patent ductus.	SMA–BFV before and 10 and 30 min after a test feed (4 mL/kg), administered 18–24 h after the last drug dose in the NPO group or 3 h after the last trophic feed in the feeding group.	No between-group difference in SMA–BFV at baseline and 30 min after the test feed. Infants in the feeding group showed significantly higher SMA–BFVs 10 min after the test feed compared to those kept NPO during pharmacological PDA closure. Indomethacin was used in 94% of the infants in each study group.

Abbreviations: BFV: Blood flow velocity; GA: Gestational age; IQR: Interquartile range; NPO: Nihil per os; (hs) PDA: (hemodynamically significant) Patent ductus arteriosus; SMA: Superior mesenteric artery; SCOR: Splanchnic–cerebral oxygen ratio; PI: Pulsatility index; RVR: Relative vascular resistance.

**Table 2:** Association between the presence of a patent ductus arteriosus (PDA) and gastrointestinal outcomes in preterm infants.

Authors	Study Population	PDA Definition	Outcome	Results
Patole et al. 2007 [31]	Preterm infants with hs-PDA (*n* = 98, median GA 25 [IQR 24–26] weeks; 78/98 received indomethacin), non-significant PDA (*n* = 30, median GA 25.5 [IQR 24–27] weeks) and no PDA (*n* = 124, median GA 27 [IQR 26–28] weeks).	hs-PDA: Pulsatile trans-ductal shunt pattern with low end-diastolic velocity (<1 m/s), left atrium-to-aortic root (LA/Ao) ratio > 1.4 or a ductal diameter > 1.5 mm. If echocardiogram was unavailable, PDA was labeled as significant on a clinical basis (systolic murmur, bounding pulses, wide pulse pressure, hyperdynamic precordium, pulmonary plethora on chest radiograph).	Days of nihil per os (NPO) and of total parenteral nutrition, age at starting feed and at full enteral feeding (FEF, enteral intakes ≥ 150 mL/kg/day) (days), days to achieve FEF, NEC (Bell’s stage ≥ 2).	Significantly longer duration of NPO and TPN periods (*p* < 0.001); significantly older age at starting feeds (*p* < 0.001) and at FEF achievement (*p* < 0.001); significantly longer time to FEF achievement (*p* < 0.001). Adjusted odds ratio (OR) for time to FEF achievement in infants with hs-PDA alone compared to no PDA: 2.80, 95% CI 1.35–5.81 (*p* = 0.006); in infants with hs-PDA and sepsis: OR 5.15, 95% CI 2.13–12.45, (*p* < 0.001). No association between NEC development and hs-PDA.
Corvaglia et al. 2014 [28]	Registry-based cohort of very low birth weight preterm infants (*n* = 1864, mean GA 29.1 ± 2.7 weeks; infants with PDA during the first week: *n* = 716).	Ultrasound evidence of a patent ductus.	Days to achieve FEF (150 mL/kg/day).	PDA persistence during the first week of life is a strong independent predictor for a longer time to FEF achievement: OR 1.276, 95% CI 1.198–1.358 (*p* < 0.001).
Havranek et al. 2015 [53]	Preterm infants with birth weight <1000 g, aged 5–7 days, with large (*n* = 9, mean GA 25.1 ± 1.3 weeks), moderate (*n* = 13, mean GA 25.8 ± 1.4 weeks), or small/no PDA (*n* = 16, mean GA 26.5 ± 1.4 weeks), not treated in the prior 24 h.	Large PDA: Ductal diameter = to-left pulmonary artery (PDA/LPA) ratio ≥ 1; moderate PDA: PDA/LPA ratio 0.5–0.9; small PDA: PDA/LPA ratio < 0.5.	Days to achieve FEF (150 mL/kg/day); incidence of NEC (Bell’s stage > 2) and death secondary to NEC.	Infants with a large PDA group reached full enteral feeding later (*p* = 0.02) and, despite that NEC rates did not differ among the study groups, had a higher incidence of death secondary to NEC (*p* = 0.04) compared to those with moderate and no/small PDA.
Martini et al. 2019 [54]	Preterm infants < 32 weeks’ gestation with hs-PDA (*n* = 11, median GA 29.4 (IQR 27.4–30.4) weeks), restrictive PDA (*n* = 11, median GA 29.5 (IQR 28.7–30.7) weeks), and with no PDA evidence (*n* = 28, median GA 29.7 (IQR 27.7–31) weeks) receiving enteral feeds within the first 72 h of life, irrespective of PDA status.	Pulsatile/hs-PDA: Pulsatile trans-ductal shunt pattern and left atrium-to-aortic root (LA/Ao) ratio ≥ 1.5; restrictive PDA: Restrictive trans-ductal shunt pattern and LA/Ao ratio < 1.5	Days to achieve FEF (150 mL/kg/day); incidence of NEC (Bell’s stage > 2), SIP, feeding intolerance (enteral feeding withholding for ≥ 24 h due to the presence of ≥2 among absent bowel sounds, abdominal distension, bloody stools, persistent bilious or bloody gastric residuals, residual volume > 2 mL/kg or >50% of previous feed volume).	No significant difference in the number of days to achieve FEF or in the incidence of NEC, SIP, and feeding intolerance between the study groups.
Clyman et al. 2013 [59]	Preterm infants < 31 weeks’ gestation (*n*= 177, mean GA 26.3 ± 1.9 weeks) receiving pharmacological treatment (indomethacin or ibuprofen) for PDA. Randomized to receive enteral intakes of 15 mL/kg/day (*n* = 81; indomethacin = 80%, ibuprofen = 20%) or to be kept NPO (*n* = 96; indomethacin = 75%, ibuprofen = 25%) during the treatment period.	Ultrasound evidence of a patent ductus requiring pharmacological closure according to the infants’ clinical care teams.	Days at central venous lines removal; incidence of NEC (Bell’s stage > 2) and SIP; difference between the actual and the ideal * number of days to reach enteral intakes of 120 mL/kg/day.	Fed infants required fewer days to reach the feeding volume endpoint compared to those kept NPO during pharmacological PDA closure. No difference in the age at which central venous lines were removed or in the incidence of NEC or SIP.
Louis et al. 2016 [60]	Preterm infants with hs-PDA undergoing different feeding regimens during indomethacin treatment: Nihil per os (NPO, *n* = 229, mean GA 26.3 ± 1.8 weeks); enteral intakes ≤ 60 mL/kg/day (*n* = 142, mean GA 26.1 ± 1.8 weeks); enteral intakes > 60 mL/kg/day (*n* = 44, mean GA 27 ± 2 weeks).	Ultrasound evidence of a patent ductus.	Time to reach enteral intakes of 120 and 160 mL/kg/day; incidence of NEC (Bell’s stage ≥ 2a); feeding intolerance (at least 2 consecutive gastric aspirates > 50% of previous feed volume or any bilious or bloody aspirate) during indomethacin treatment; duration of total parenteral nutrition.	No difference in NEC incidence was observed among the study groups. Infants kept NPO during indomethacin treatment had significantly lower rates of feeding intolerance compared to infants fed ≤ 60 mL/kg/day (*p* = 0.04). Infants fed > 60 mL/kg/day during indomethacin treatment required a significantly lower number of days to achieve enteral intakes of 120 and 160 mL/kg/day compared to those fed ≤ 60 mL/kg/day (*p* = 0.01 and *p* = 0.03, respectively) or kept NPO (*p* < 0.01 and *p* = 0.01, respectively). Infants fed ≤ 60 mL/kg/day during indomethacin treatment required a significantly lower number of days to achieve enteral intakes of 120 (*p* = 0.02), but not of 160 mL/kg/day, compared to those kept NPO.

Abbreviations: GA: Gestational age; IQR: Interquartile range; NEC: Necrotizing enterocolitis; NPO: Nihil per os; (hs) PDA: (hemodynamically significant) Patent ductus arteriosus; hs-PDA: Hemodynamically significant patent ductus arteriosus; SIP: Spontaneous intestinal perforation; FEF: Full enteral feeding; OR: Odds ratio; CI: Confidence interval; TPN: total parenteral nutrition. * Based on the assumption that the infant would be kept NPO during the drug administration.
